# Endovascular navigation in patients: vessel-based registration of electromagnetic tracking to preoperative images

**DOI:** 10.3389/fradi.2024.1320535

**Published:** 2024-01-23

**Authors:** Erik Nypan, Geir Arne Tangen, Reidar Brekken, Petter Aadahl, Frode Manstad-Hulaas

**Affiliations:** ^1^ Institute of Circulation and Medical Imaging, Faculty of Medicine and Health Sciences, Norwegian University of Science and Technology (NTNU), Trondheim, Norway; ^2^ Norwegian National Research Centre for Minimally Invasive and Image-Guided Diagnostics and Therapy, St. Olavs Hospital, Trondheim, Norway; ^3^ Department of Health Research, SINTEF Digital, Trondheim, Norway; ^4^ Department of Cardiothoracic Anesthesia and Intensive Care, St. Olavs Hospital, Trondheim, Norway; ^5^ Department of Radiology, St. Olavs Hospital, Trondheim, Norway

**Keywords:** endovascular navigation, registration accuracy, electromagnetic tracking, aortic aneurysm, preoperative CT

## Abstract

Electromagnetic tracking of instruments combined with preoperative images can supplement fluoroscopy for guiding endovascular aortic repair (EVAR). The aim of this study was to evaluate the in-vivo accuracy of a vessel-based registration algorithm for matching electromagnetically tracked positions of an endovascular instrument to preoperative computed tomography angiography. Five patients undergoing elective EVAR were included, and a clinically available semi-automatic 3D–3D registration algorithm, based on similarity measures computed over the entire image, was used for reference. Accuracy was reported as target registration error (TRE) evaluated in manually selected anatomic landmarks on bony structures, placed close to the volume-of-interest. The median TRE was 8.2 mm (range: 7.1 mm to 16.1 mm) for the vessel-based registration algorithm, compared to 2.2 mm (range: 1.8 mm to 3.7 mm) for the reference algorithm. This illustrates that registration based on intraoperative electromagnetic tracking is feasible, but the accuracy must be improved before clinical use.

## Introduction

Endovascular aortic repair (EVAR) is routinely performed under fluoroscopic guidance. To reduce radiation and contrast dose, electromagnetic (EM) tracking of instruments with registration to preoperative computed tomography angiography (CTA) can be an appealing supplement. EM tracking has been demonstrated in feasibility studies, including in-situ fenestration of stent graft and cannulation of branch vessels (1–7). Most reported results are from *in vitro* phantom experiments, some from in-vivo animal studies, but very limited from patients.

A simple and accurate algorithm for registration between EM coordinates and preoperative images is a key technology for enabling EM based navigation. While most studies have applied fiducial based registration, a drawback of this technique is that fiducials must be visible in the preoperative images, which requires short time between imaging and procedure. Further, the fiducials can be far from the region of interest and subject to deformation and thereby decreased accuracy (8).

A registration algorithm based on automatic matching between electromagnetically tracked instrument positions and the aorta centerline extracted from preoperative CTA has previously been evaluated in a phantom model (9), and in-vivo in swine (10). The aim of this study was to examine the accuracy in patients undergoing EVAR and compare it to a clinically available semi-automatic registration algorithm for reference.

## Methods

Five patients undergoing elective EVAR for infrarenal abdominal aortic aneurysm were included after informed consent. The study protocol was approved by the Regional Committees for Medical and Health Research Ethics (REK 2016/533) and registered on clinicaltrials.gov (NCT03116880).

Preoperative CTA had already been routinely acquired for clinical planning before the procedure (Siemens Sensation 64 CT-scanner, 512 × 512 pixels, 0.55 mm/pix, slice distance 0.7 mm, CTA aorta abdomen protocol). For study purpose, the CTA was exported from the picture archiving system (PACS) in DICOM format.

The study was performed in a hybrid operating room equipped with an Artis Zeego (Siemens Healthineers, Germany) able to acquire intraoperative cone-beam computed tomography (CBCT, Siemens DynaCT, 512 × 512 pixels, 0.49 mm/pix, slice distance 0.49 mm). The navigation system consisted of an EM tracking system (Aurora, Northern Digital Inc., Germany) connected to the research navigation platform CustusX (11) with the field generator mounted underneath the operating table.

Before the procedure, two custom made registration plates were attached to the back of the patients at the level of the renal arteries for evaluation purpose. Each registration plate contained seven 0.8 mm radiopaque spherical Tantalum markers (Tilly Medical Products AB, Sweden) and two 5-Degrees of Freedom (DoF) EM-sensors. A non-contrast CBCT including the two registration plates was acquired before sterile draping, after which the patients were not moved.

After establishing vascular access in both femoral arteries, a 6-French pigtail catheter (Boston Scientific, MA, US) was advanced under fluoroscopic guidance into the proximal segment of the descending aorta. An EM catheter sensor (Aurora, Northern Digital Inc., 5 DoF Flex tube) was then advanced as far as possible through the working channel of the pigtail catheter. In case the EM sensor could not be sufficiently advanced to acquire complete position data from the juxta-renal segment of the aorta, the patient was excluded without any additional attempts. Next, the pigtail catheter was slowly withdrawn with a rotating motion until it reached the introducer, while the position of the EM sensor inside was recorded with a frequency of 40 Hz, generating a point cloud of sampled position data which was stored for postprocessing. This procedure was repeated on the contralateral side before the EVAR procedure continued as normal, with conventional fluoroscopy and angiography guidance.

### Centerline registration method

**Figure 1:**
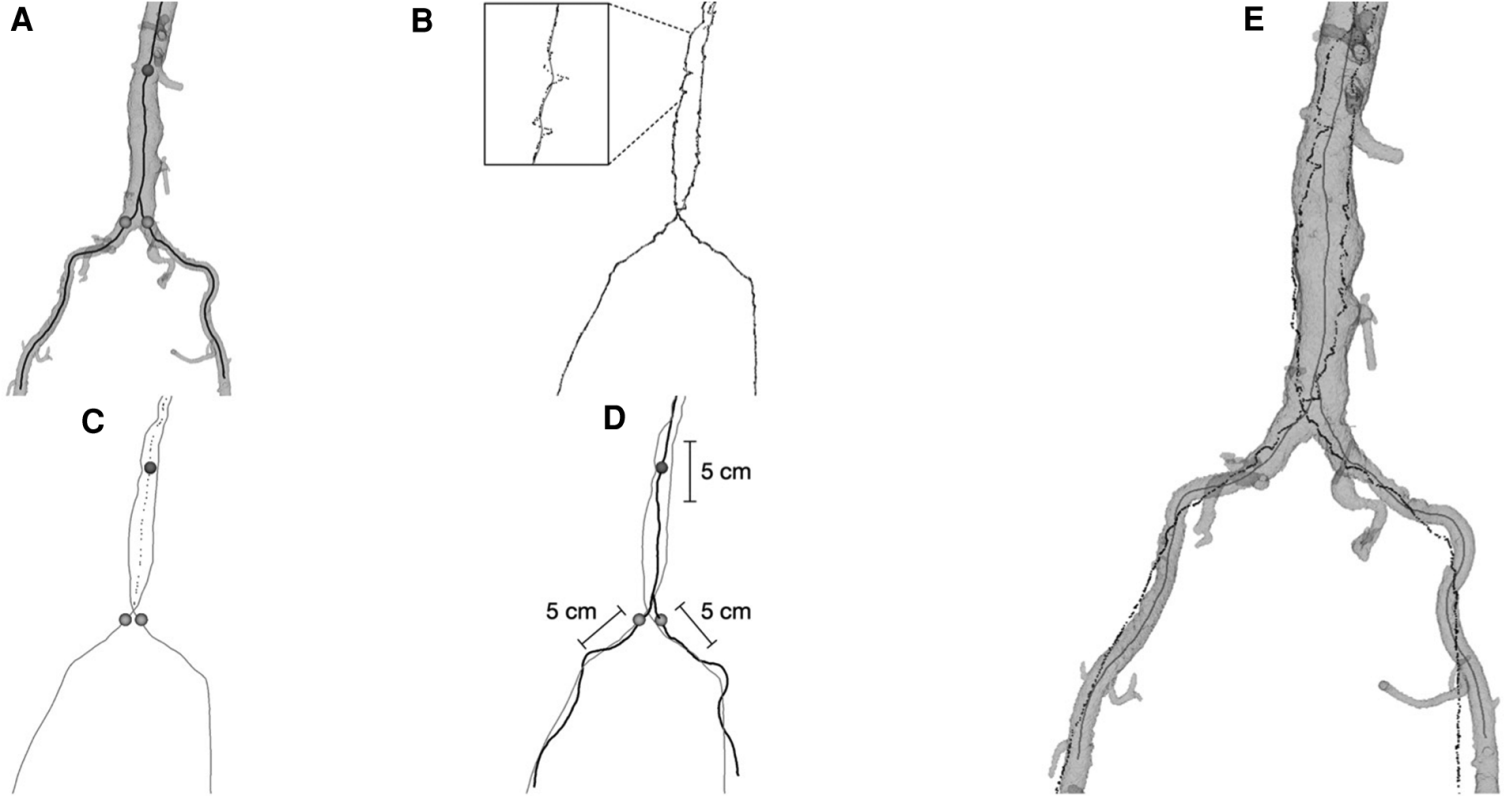
(**A**) the segmented aorta model with centerlines from CTA and the three points (sphere markings) on the centerlines used for initial landmark registration. (**B**) The point-clouds of recorded position data with processed midline (shown magnified). (**C**) The position data midlines and the processed combined midline for the aorta part (dotted line) together with the three extracted points for initial registration. (**D**) Centerlines from CTA (black) together with midlines from position data (grey), indicating the 5 cm intervals of centerlines from CTA used in the final registration. (**E**) Final registration result, aorta model with centerlines (grey) together with registered position data (black).

The in-house centerline registration algorithm consisted of a landmark-based registration used for initialization, followed by a rigid-body iterative closest point (ICP) algorithm (12) automatically matching the EM tracked positions to selected segments of the preoperative centerlines. For the first part, the iliac ostia and a point in the aorta close to the renal artery ostia were manually marked in the CTA images and corresponding feature points were automatically identified in the EM position data. For the ICP algorithm, segments of the centerlines were automatically selected from the juxta-renal aorta and upper part of the iliac arteries from the CTA, each with a length of 5 cm (Figure 1). More information about the registration method could also be found in Nypan et al. (9, 10).

The lumen and centerline of the aorta and iliac vessels were segmented from the CTA using the open-source toolkits ITK-SNAP (version 3.6.0) (13), and VMTK (version 1.3) (14). The registration method was implemented as a plugin module to the CustusX platform (11) based on the ICP algorithm provided by the open-source Insight Toolkit platform (ITK, itk.org).

### Reference algorithm

A semi-automatic registration algorithm (Siemens Multimodality Workplace, Siemens Healthineers, Germany) for registering preoperative CTA to intraoperative CBCT was used as a reference. A radiologist with more than ten years' experience performed the registration, and manual corrections were performed with intent to register the aorta optimally in cases where the registration was insufficient, e.g., if aortic calcifications were misaligned or the wrong level of vertebral bodies had been registered.

### Evaluation

Anatomic landmarks were used to evaluate registration accuracy (Table 1). As the landmarks were defined by the volume covered by the CBCT, landmarks were first identified in the CBCT and thereafter correspondingly in the CTA. The manual markings were performed by an experienced radiologist using the open-source DICOM reader Horos (version 3.3.6, horosproject.org). To verify the integrity of the manual annotations, the fiducial registration error (FRE) was calculated for each patient as the root mean square (RMS) distance between corresponding landmarks after applying a rigid-body point-based registration.

**Table 1:** Anatomic landmarks for accuracy evaluation.

Landmark description relative to the CBCT volume
1. The most lateral point of the left transverse process of the most cranial completely imaged vertebra
2. The most posterior point of the spinous process of the most cranial completely imaged vertebra
3. The most lateral point of the right transverse process of the most caudal completely imaged vertebra
4. The most anterior point of the vertebral body of the most caudal completely imaged vertebra
5. The most anterior point of the lower vertebral body of the second most caudal completely imaged vertebra
6. The most anterior point of the lower vertebral body of the first most caudal completely imaged vertebra

The TRE of the reference registration between CBCT and CTA was calculated as the RMS based on the 3D Euclidean distance for each of the six landmark pairs in CTA and the registered CBCT volume.

**Figure 2:**
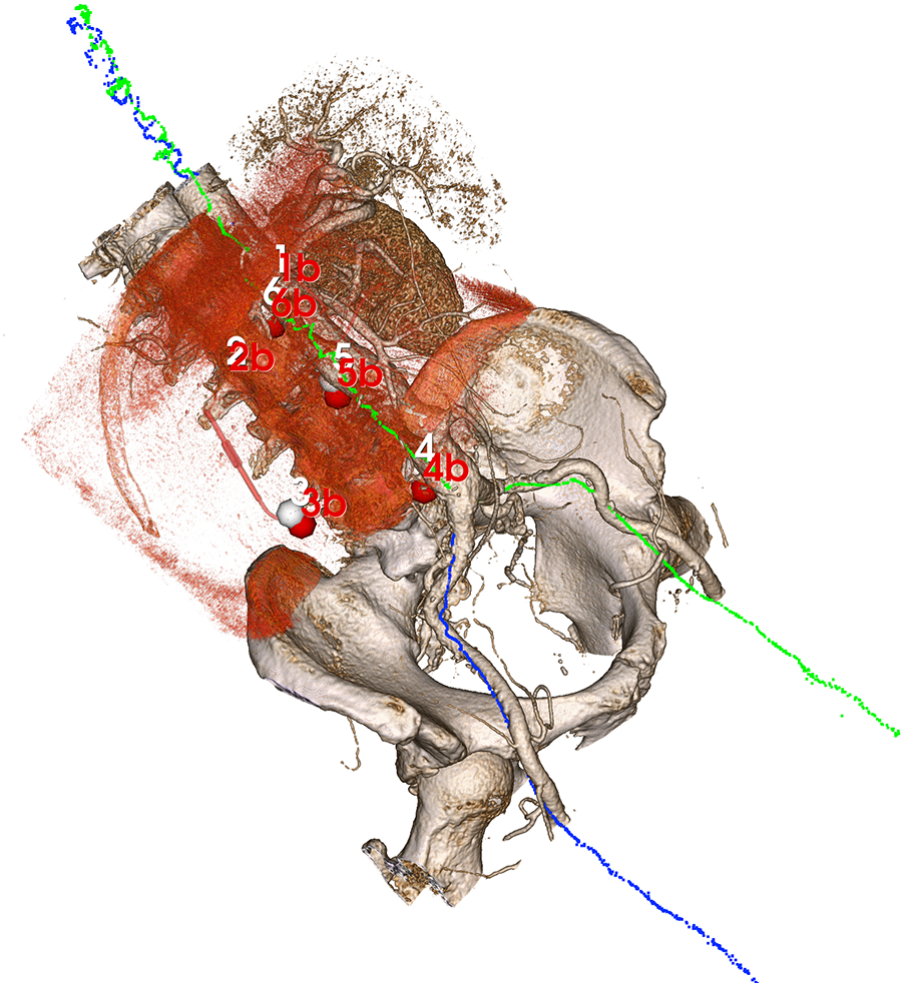
Volume rendering of the CTA after centerline registration with the blue and green point clouds illustrating electromagnetically sampled positions during pullback through the aorta and into the right and left iliac arteries, respectively. Since the sampled positions are registered to the CBCT (volume rendered in red tone) through the registration plates, an indirect registration between the CTA and CBCT is obtained from the centerline algorithm, allowing for an evaluation of target registration error in the six manual landmarks (CTA points in white, CBCT point in red).

To use the same landmarks also for evaluating the centerline registration, the position coordinates of the landmarks from CBCT were transformed into the EM coordinate system using the registration plates. The landmarks from CTA were transformed into the same EM coordinate system by applying the centerline registration. The TRE could then be calculated as the RMS for the six landmark pairs in the same way as for the reference registration (Figure 2).

TRE was calculated first by including all six landmarks for each patient, and then by including only the three landmarks closest to the aorta.

## Results

The median FRE for the manual annotations was 1.8 mm (range: 1.1 mm to 2.7 mm). The median TRE of the centerline method was 8.2 mm (range: 7.1 mm to 16.1 mm), compared to 2.2 mm (range: 1.8 mm to 3.7 mm) for the reference method, with only slight improvements when evaluated only for the three landmarks closest to the aorta (8.0 mm and 1.9 mm, respectively). For EVAR procedure, the accuracy in the cranio-caudal direction is the most important. However, we did not find any predominant direction vector when analyzing for the sideways and craniocaudal deviation separately.

The results for all five patients are shown in Table 2.

**Table 2:** Target registration errors calculated over all six landmarks and for the three landmarks closest to the aorta in parenthesis (landmark 4–6). All numbers are RMS values in mm.

	Landmark registration	Reference algorithm	Centerline registration
Case 1	1.8	2.2 (1.9)	12.9 (8.0)
Case 2	1.6	2.1 (2.6)	8.2 (9.0)
Case 3	1.1	1.8 (1.8)	16.1 (14.5)
Case 4	2.1	2.6 (0.9)	7.4 (5.0)
Case 5	2.7	3.7 (4.5)	7.1 (4.6)
Median	1.8	2.2 (1.9)	8.2 (8.0)

## Discussion

The aim of this study was to examine the accuracy of a vessel-based centerline registration algorithm and compare it to a clinically available reference in patients undergoing EVAR. The method could contribute to simplify use of image-fusion, and to reduce intraoperative radiation and contrast doses. Although the results suggested that registration of electromagnetic position data from the aorta to preoperative images can be feasible, the registration accuracy was inferior to that of the reference. The median TRE of 8.2 mm is high compared to results from *in vitro* phantom studies (3.75 mm and 1.3 mm) (9, 15), which highlights both added complexity in clinical settings and the need for accuracy evaluations in clinical trials.

A TRE lower than 5 mm has previously been proposed as an acceptable registration error (16). When evaluating TRE for the three target landmarks closest to the aorta, the value was lower than 5 mm in only two out of five cases for the centerline algorithm, whereas it was lower in all five cases for the reference method. Higher thresholds could possibly be acceptable in some cases. In addition to numerical accuracy, success rate of navigation should therefore also be evaluated for specific clinical tasks.

Several sources of inaccuracy may be identified. Since the preoperative images were acquired one to three months in advance of the surgery, and positioning in the CT-scanner and on the operating table differs, deformation is inevitable. This inaccuracy was diminished for the reference method since manual corrections were made if the aorta was mismatched. Further, our approach assumes that the catheter circles around the vessel centerline. However, the catheter may instead follow a pathway along the vessel wall which could lead to inaccuracies when applying the centerline algorithm, especially for larger vessels. Disturbances in the electromagnetic field could further compromise the accuracy of the EM tracking.

The time required to run the semi-automatic reference registration algorithm during the procedure was around 5 min. The centerline algorithm completed a registration in less than 1 min after the catheter pull-down sequence. In future development of this registration algorithm, we will investigate the real-time potential of the method. The software can continuously sample the catheter position during the procedure and the registration algorithm can work unattended as a background task to improve the registration accuracy during the clinical procedure.

The pigtail catheter was chosen to ensure a distance from the aortic wall to the sampled position data to align better with the centerline of the blood vessels. Choosing another catheter could possibly influence the distribution of the sampled position data and consequently the result of the registration.

A study limitation was the need for registration plates to transform the positions of the anatomical landmarks from CBCT image coordinates to the EM coordinate system for evaluation of the centerline algorithm. This added an extra source of error compared to the reference method. Although the plate registration error is small, dislocation of the registration plates during the procedure will inevitably degrade accuracy. In this study, the registration plates were however needed for the accuracy calculation only, not for the centerline registration. An implementation of the centerline registration in a clinical procedure is therefore not dependent on the registration plates.

As described in the Methods section, the EM sensor was advanced as far as possible into the working channel of the pigtail catheter after the catheter was inserted in the descending aorta. The approach was chosen to secure sterile instrumentation, but unfortunately resulted in missing data from eleven patients where the sensor could not be sufficiently advanced to acquire complete position data from the juxta-renal segment of the aorta. For further clinical research, approved endovascular instruments with embedded EM sensors are therefore warranted.

Using anatomical features, such as the vessel centerlines, has the potential to simplify the registration by avoiding the need for preoperative images containing fiducials or acquisition of additional CBCT for 3D–3D registration. Also, as deformation inevitably occurs during EVAR, an acceptable registration at the start of the procedure may become inaccurate after the introduction of instruments in the aorta (e.g., stiff wires or stent graft delivery system). If the position of endovascular instruments is continuously tracked during the procedure, the centerline registration algorithm can in principle run in the background to update the registration. This may lead to a more accurate registration throughout the procedure.

In summary, this study suggests that a vessel-based centerline registration algorithm could be used for matching preoperative CTA images to tracked position data from endovascular tools inside the blood vessels in patients, but the accuracy needs to be improved before clinical use.

## Data Availability

The raw data supporting the conclusions of this article will be made available by the authors, without undue reservation.

## References

[1] Oliny A Goel VR Rebet A Hengstum S van Magistrelli F Grandt A Branched endovascular thoracoabdominal aneurysm repair under electromagnetic guidance in an in vitro model. J Endovasc Ther. (2023) 30:786–91. 10.1177/15266028231162260 36942690

[2] Sieren MM Jäckle S Eixmann T Schulz-Hildebrandt H Matysiak F Preuss M Radiation-free thoracic endovascular aneurysm repair with fiberoptic and electromagnetic guidance: a phantom study. J Vasc Interv Radiol. (2022) 33:384–91.e7. 10.1016/j.jvir.2021.12.025 34958860

[3] West K Al-Nimer S Goel VR Yanof JH Hanlon AT Weunski CJ Three-dimensional holographic guidance, navigation, and control (3D-GNC) for endograft positioning in porcine aorta: feasibility comparison with 2-dimensional x-ray fluoroscopy. J Endovasc Ther. (2021) 28:796–803. 10.1177/15266028211025026 34142900 PMC8922995

[4] Condino S Piazza R Viglialoro RM Mocellin DM Turini G Berchiolli RN Novel em guided endovascular instrumentation for in situ endograft fenestration. IEEE J Transl Eng Health Med. (2020) 8:1–8. 10.1109/JTEHM.2020.2973973 PMC7082146 32219042

[5] Penzkofer T Na HS Isfort P Wilkmann C Osterhues S Besting A Electromagnetically navigated in situ fenestration of aortic stent grafts: pilot animal study of a novel fenestrated EVAR approach. Cardiovasc Intervent Radiol. (2018) 41:170–6. 10.1007/s00270-017-1769-z 28821949

[6] Schwein A Kramer B Chinnadurai P Virmani N Walker S O'Malley M Electromagnetic tracking of flexible robotic catheters enables “assisted navigation” and brings automation to endovascular navigation in an in vitro study. J Vasc Surg. (2018) 67:1274–81. 10.1016/j.jvs.2017.01.072 28583735

[7] Manstad-Hulaas F Tangen GA Dahl T Hernes TAN Aadahl P. Three-dimensional electromagnetic navigation vs. fluoroscopy for endovascular aneurysm repair: a prospective feasibility study in patients. J Endovasc Ther. (2012) 19:70–8. 10.1583/11-3557.1 22313205

[8] Fitzpatrick JM West JB Maurer CR Jr. Predicting error in rigid-body point-based registration. IEEE Trans Med Imaging. (1998) 17:694–702. 10.1109/42.736021 9874293

[9] Nypan E Tangen GA Manstad-Hulaas F Brekken R. Vessel-based rigid registration for endovascular therapy of the abdominal aorta. Minim Invasive Ther Allied Technol. (2019) 28:127–33. 10.1080/13645706.2019.1575240 30810444

[10] Nypan E Tangen GA Brekken R Manstad-Hulaas F. A steerable and electromagnetically tracked catheter: navigation performance compared with image fusion in a swine model. J Endovasc Ther. (2022). 10.1177/15266028221123434 [Epub ahead of print]. 36121010 PMC10938482

[11] Askeland C Solberg OV Bakeng JBL Reinertsen I Tangen GA Hofstad EF Custusx: an open-source research platform for image-guided therapy. Int J Comput Ass Rad. (2016) 11:505–19. 10.1007/s11548-015-1292-0 PMC4819973 26410841

[12] Besl PJ McKay ND. A method for registration of 3-D shapes. IEEE Trans Pattern Anal. (1992) 14:239–56. 10.1109/34.121791

[13] Yushkevich PA Piven J Hazlett HC Smith RG Ho S Gee JC User-guided 3D active contour segmentation of anatomical structures: significantly improved efficiency and reliability. Neuroimage. (2006) 31:1116–28. 10.1016/j.neuroimage.2006.01.015 16545965

[14] Antiga L Piccinelli M Botti L Ene-Iordache B Remuzzi A Steinman DA. An image-based modeling framework for patient-specific computational hemodynamics. Med Bio Eng Comput. (2008) 46:1097–112. 10.1007/s11517-008-0420-1 19002516

[15] de Lambert A Esneault S Lucas A Haigron P Cinquin P Magne JL. Electromagnetic tracking for registration and navigation in endovascular aneurysm repair: a phantom study. Eur J Vasc Endovasc Surg. (2012) 43:684–9. 10.1016/j.ejvs.2012.03.007 22487781

[16] Manstad-Hulaas F Tangen GA Demirci S Pfister M Lydersen S Nagelhus Hernes TA. Endovascular image-guided navigation: validation of two volume-volume registration algorithms. Minim Invasive Ther Allied Technol. (2011) 20:282–9. 10.3109/13645706.2010.536244 21091381

